# Association between iron supplementation and the presence of diarrhoea in Peruvian children aged 6–59 months: analysis of the database of the Demographic and Family Health Survey in Peru (DHS, Peru), years 2009–2019

**DOI:** 10.1017/S1368980021004808

**Published:** 2022-10

**Authors:** Valeria Janice Valverde-Bruffau, Kyle Steenland, Gustavo F Gonzales

**Affiliations:** 1 High Altitude Research Institute, Universidad Peruana Cayetano Heredia, 430 Honorio Delgado Av. San Martin de Porres, Lima 15102, Peru; 2 Laboratories of Investigation and Development (LID), Department of Biological and Physiological Sciences, Faculty of Sciences and Philosophy, Universidad Peruana Cayetano Heredia, Lima, Peru; 3 Gangarosa Department of Environmental Health, Rollins School of Public Health, Emory University, Atlanta, GA, USA

**Keywords:** Anaemia, Diarrhoea, Iron supplement, Drinking water, Sewage

## Abstract

**Introduction:**

According to the WHO, anaemia is a severe public health problem when the prevalence is ≥ 40 %. In 2019, in Peru, 40·1 % of children (aged 6 to 35 months) are diagnosed as anaemic. This is a concern since, despite the efforts of the governments to reduce the prevalence, the problem has stagnated since 2011. The treatment applied to deal with anaemia is Fe supplementation. Although Fe is essential for cell function, an excess can produce adverse responses, such as gut inflammation affecting microbiota and resulting in diarrhoeic episodes.

**Objective:**

To determine the association between diarrhoea and Fe supplementation in children with and without anaemia, controlling for different socio-demographic variables.

**Design:**

We conducted via logistic regression to obtain diarrhoea prevalence ratios (PR), adjusted by age, sex, geographic region, water and sanitation service, and rurality. The survey asked for recent episodes of diarrhoea during the last 7 d; similarly, after the consumption of Fe supplements during the last 12 months before the survey.

**Setting:**

Peru.

**Participants:**

The Demographic and Family Health Survey (DHS) is conducted annually at home among 14 202 children on average (2009–2019).

**Results:**

Fe supplementation in the last 7 d (PR = 1·09) or the last 12 months (PR = 1·19) (*P* < 0·0001) was associated with an increased risk of diarrhoea. The same association was observed between Fe supplementation and the presence of anaemia.

**Conclusions:**

Fe supplementation is associated with diarrhoea and overuse in children should be avoided.

Anaemia is a condition that affects 27 % of the world’s population worldwide^(1)^. According to 2015 reports based on a 2011 study, the (WHO reported that anaemia affects 800 million women and children. The highest prevalence of anaemia was in children (42·6 %, 95 % CI (37, 47))^(2)^.

Worldwide, the prevalence of anaemia has decreased between 2000 and 2010 and has since remained steady^(3)^. According to World Bank data, 32·5 % of children between 6 and 59 months of age in Peru were diagnosed as anaemic in 2016^(3)^.

Anaemia is a disorder in which the number of erythrocytes (and therefore the oxygen-carrying capacity of the blood) is insufficient to meet the needs of the body^(4)^. Although an automated blood count assessment is recommended by the WHO in its latest guide, for places where there is no access to automated counters, the use of Hb to define anaemia is still recommended^(5)^. The WHO considers a diagnosis of anaemia when the Hb concentration is below 11·0 g per decilitre (g/dl) in children aged 6 to 59 months. An adjustment of the Hb cut-off to define anaemia since 1000 m of altitude has also been recommended^(4)^.

Anaemia is pathologically diverse and often multifactorial. The WHO estimates that Fe deficiency accounts for 50 % of anaemia cases globally^(2)^; however, recent studies show that the fraction of anaemia attributable to Fe deficiency may be less than previously estimated^(6–7)^. The second most important cause of anaemia is inflammation (infectious or non-infectious) and it is estimated that it occurs in 42 % of cases, although this figure may vary among populations^(6)^.

Using the WHO cut-off point to define childhood anaemia (Hb < 11 g/dl) and the correction for altitude proposed by WHO taken into account, in the year 2005, the number of anaemic children^(8–10)^.

Guidelines from the Ministry of Health in Peru recommend Fe supplements for all children aged 6–59 months, whether or not they are anaemic. The criterion is based on the existence of Fe deficiency cases without anaemia, and that anaemia represents the most severe state of such deficiency. For this reason, the government suggests supplementing with Fe to all children aimed to prevent children with a Fe deficiency from becoming anaemic. However, not all cases of anaemia are attributable to Fe deficiency^(7)^.

In 2018, Peruvian departments located in higher altitudes such as Puno and Cusco, at 3400 and 3800 m above sea level, respectively, showed an even further increased prevalence of anaemia despite an intense campaign by the Peruvian government to reduce the incidence rates^(10)^.

The programmes are based on new regulations aimed to decrease anaemia rates via mandatory Fe supplementation in children over 4 months of age. Supplementation initiates with drops of ferrous sulphate and progressively includes other medications such as micronutrients or pills, both for children diagnosed with anaemia (therapeutic) and without anaemia (preventive)^(11)^. The Peruvian government also approved Law 28314 on 9 July 2004, for the mandatory fortification of wheat flour with Fe among other four micronutrients. And, on 18 August 2021, Law 31348 for the mandatory fortification of rice with Fe was approved.

The concern arises about the effect of Fe excess received by non-anaemic children. Fe not entering into the duodenal enterocytes, or Fe stored in ferritin that is removed upon enterocyte desquamation, passes into the large intestine, generating an Fe excess^(12–13)^.

The Fe concentration reaching the colon will be higher since the diet Fe amount is higher^(14)^ and may affect the microbiome composition. This alteration, known as dysbiosis, is responsible for the adverse effects observed as inflammation^(15–17)^ and diarrhoea^(18–20)^.

It has been shown that the Fe content of the diet is related to the number of enterobacteria and lactobacillus in the mouse intestine^(21)^. In addition, Fe administration at the required standard dose could decrease the relative abundance of lactobacilli and potentially increase susceptibility to bacterial infection in the gut microbiota of Fe-sufficient infants^(22)^.

The excessive amount of unabsorbed Fe in the colonic lumen favours greater inflammatory activity^(23)^, which in turn increases serum hepcidin levels. Hepcidin is the hormone responsible for Fe availability in the body^(24)^. Its increase will result in lower availability of Fe in tissues for the body cell activities, including erythropoiesis^(14)^, resulting in inflammatory anaemia which would not respond to Fe supplementation.

In children, the microbiota is developing, and continuous gut exposure to excess Fe concentrations can affect the relationship between commensal and pathogenic bacteria, increasing the proliferation of the latter, causing diarrhoea as a result^(25)^.

Middle- and lower-income countries are characterised by a higher infections prevalence and a lack of sanitary services (drinking water and sanitation). These factors are associated with an increased risk of diarrhoea^(26)^ and may worsen the association between Fe supplementation and episodes of diarrhoea^(14)^.

This study has been designed to determine whether Fe supplementation in the population of non-anaemic and anaemic Peruvian children aged 6 to 59 months is associated with the presence of diarrhoea after controlling for age, sex, geographical region, rural or urban lifestyle, vaccination against rotavirus, access to drinking water, and access to sanitation.

## Materials and methods

### Population

Our study was a cross-sectional analysis of a secondary database from the Demographic and Family Health Survey (DHS)-Peru. The dataset used was obtained annually from 2009 to 2019. This survey used a stratified multi-stage randomised sampling design.

The survey was conducted annually by the National Institute of Statistics and Informatics (INEI), an institution of the government of Peru, in the twenty-five regions of the country, and collects information about reproductive, maternal and child health during a home visit. Homes evaluated in 1 year were not selected in the following years, so there was no risk of repeated samples. Our unit of analysis included children between 6 and 59 months of age.

The original sample included 177 248 children, of whom 162 933 were aged between 6 months and 59 months. Children with Hb values ≥3 g/dl were included in the study; values below 3 g/dl could indicate errors in the analysis or in obtaining the sample^(27)^. The final sample size for the analyses was 156 224. Of these, 79 770 were boys and 76 454 were girls.

### Variables included in models

#### Outcomes and exposure variables

Our exposure variable was Fe supplementation as a dichotomous variable. The DHS asked if the child consumed or not the Fe supplementation during a reference period of 7 d or 12 months before the survey date.

The data were presented as the percentage of children aged 6 to 35 months who have consumed the Fe supplement in the last 7 d or the last 12 months, to ensure the adequate supply of this nutrient in the diet of children, to prevent and reduce the prevalence of anaemia.

The issue of Fe supplementation has been the subject of analysis by the DHS-Peru since 2007. Since 2013, the Fe supplement comprised Fe in pills or syrup, Fe powder such as sparks or stars, Fe in drops, and another presentation. Dosage was 2–3 mg/kg/d for children aged 4–6 months and 12·5 mg elemental Fe in multimicronutrients for children aged 6–59 months. According to DHS, 70 % of children consuming Fe supplements used envelopes of multimicronutrients. Also, it shows data of Fe supplementation during the 7 d before the survey from 2013 to 2019, whereas data of Fe supplementation during the last 12 months before the survey were presented from 2015 to 2019.

Our outcome variable was diarrhoea, defined by whether the infant had recent episodes of diarrhoea in the prior 7 d, as reported by the mother^(8)^.

Anaemia was defined when Hb values were below 11 g/dl. For the different analyses, we have calculated the prevalence of anaemia without Hb correction. When anaemia was presented over time, we have calculated anaemia with and without Hb correction by altitude.

#### Potential confounding variables

The variables included in the regression models were individual child characteristics (the sex of the child, age, rotavirus vaccination and Fe supplementation), maternal characteristics (maternal education) and household factors (access to toilet facilities and sources of drinking water). Maternal education was defined as the highest level achieved among four categories: without education (0), elementary education (1), high school (2) and university (3). Access to piped water was defined as a dichotomous variable, coded yes if the child had drinking water service at home. Sewage was defined also as a dichotomous variable depending on whether the child had access or not to sewer services at home.

The community-level variables in the model included the three main regions of Peru (coast, highlands and jungle) and place of residence (urban/rural).

#### Effect modification by age

We considered age as a possible effect modifier of the association between Fe supplementation and diarrhoea by conducting separate analyses of the Fe/diarrhoea association according to age group.

There were four age groups. The first group was aged 6–11 months, the second 12–23 months, the third 24–35 months and the fourth 36–59 months.

### Ethical aspects

The dataset does not contain any information identifying the participants of the study, ensuring the confidentiality of the data. Codes were used that provide the full anonymity of the mothers and children, both of which will share the same code.

### Statistical analysis

The methodology for selecting the sample of the DHS is complex; the survey was designed to be representative of the country. Analyses required the use of weights per individual, provided by the database. This preserves the representativeness of data at a national level.

We first conducted descriptive analyses with bivariate data using the chi-squared test to determine whether there was an association between Fe supplementation and diarrhoea, and the variation according to anaemia status.

We then used a generalised linear model with log-binomial regression^(28)^ to determine the association between Fe supplementation and the prevalence of diarrhoea while controlling for potential confounders – age, water supply, access to sewage systems, sex, place of residence, region, rotavirus vaccination and the mother’s level of education, also assessing effect modification by anaemia. Results are presented as prevalence ratios (PR)^(29)^.

Additionally, multicollinearity was assessed using the variance inflation factor test. The test evaluates the correlation between each independent variable; the wealth index was highly correlated with sanitation service access and maternal education. Therefore, the wealth index was excluded from the model.

The results were considered statistically significant when *P* < 0·05. Analyses were done using the STATA v14.0 statistical package (StataCorp.).

## Results

**Fig. 1:**
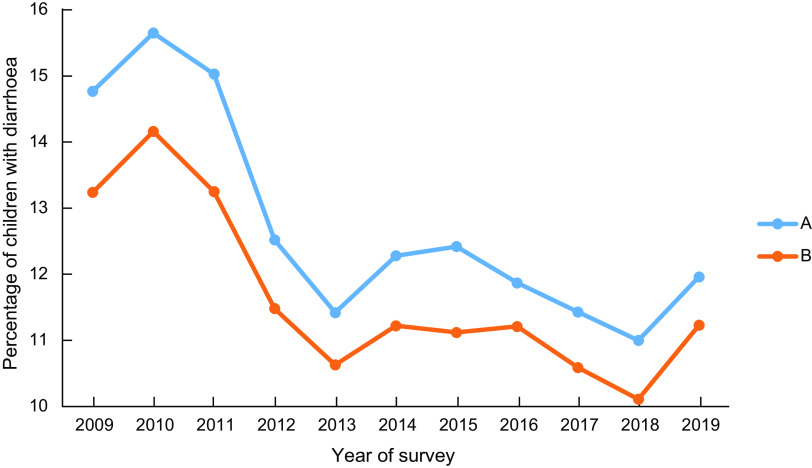
(a) The per cent of children who presented with episodes of diarrhoea by year of the survey. (b) The per cent of children who presented with episodes of diarrhoea that have potable water and sanitation service by year of survey

The frequency of Peruvian children aged 6–59 months with recent episodes of diarrhoea dropped from 2009 to 2013 and remained relatively steady through 2019 in both the total population assessed in the surveys (Fig. 1(a)) and the group of children with access to drinking water at home (Fig. 1(b)). Although the pattern through the years is the same, the prevalence of diarrhoea was lower in the group of children with drinking water at home.

Children with diarrhoea are younger, male, less likely to be anaemic, more likely to have received Fe supplementation in the last 12 months and had less access to drinking water and sewage. Their mothers have a lower level of education and resided less in urban areas and more in rural areas. They had a higher percentage of both doses of the rotavirus vaccine and were poorer. In addition, they lived more often in the highlands and jungles of Peru (Table 1).

**Table 1:** Descriptive variables in children with and without recent episodes of diarrhoea in Peru

	With diarrhoea 19 112	Without diarrhoea 126 779	*P*
Age			
Mean	26·06	33·69	< 0·0001
sd	14·06	15·42	
Male	10 347	63 987	
	54·1 %	50·5 %	< 0·0001
Per cent of anaemics	5870	26 177	
	30·7 %	20·6 %	< 0·0001
Fe supplementation (7 d)	3857	20 762	
	27·0 %	20·8 %	< 0·0001
Fe supplementation (12 months)	7877	43 060	
	64·3 %	50·0 %	< 0·0001
Piped water	14 370	101 568	
	76·9 %	81·3 %	< 0·0001
Sanitation service	9527	70 916	
	51·0 %	56·8 %	< 0·0001
Maternal education			
No education	373	3127	
	1·9 %	2·5 %	< 0·0001
Elementary	4929	32 396	
	25·8 %	25·5 %	< 0·0001
Middle school	12 030	76 855	
	62·9 %	60·6 %	< 0·0001
College	1780	14 400	
	9·3 %	11·4 %	< 0·0001
Residence			
Urban	12 187	83 373	
	63·8 %	65·8 %	< 0·0001
Rural	6925	43 406	
	36·2 %	34·2 %	< 0·0001
Region			
Coast	7447	60 267	
	38·9 %	47·5 %	< 0·0001
Highlands	6377	40 528	
	33·4 %	31·9 %	< 0·0001
Jungle	5288	25 984	
	27·7 %	20·5 %	< 0·0001
Rotavirus vaccine with vaccine (both doses)	13 012	82 243	
	68·2 %	64·9 %	< 0·0001
Wealth index			
Very poor	5903	35 995	
	30·9 %	28·4 %	< 0·0001
Poor	5758	33 343	
	30·1 %	26·3 %	< 0·0001
Medium	3823	25 929	
	20 %	20·4 %	< 0·0001
Rich	2382	18 907	
	12·5 %	14·9 %	< 0·0001
Very rich	1246	12 605	
	6·5 %	9·9 %	< 0·0001

Anaemia was defined without correction of Hb by altitude.

**Fig. 2:**
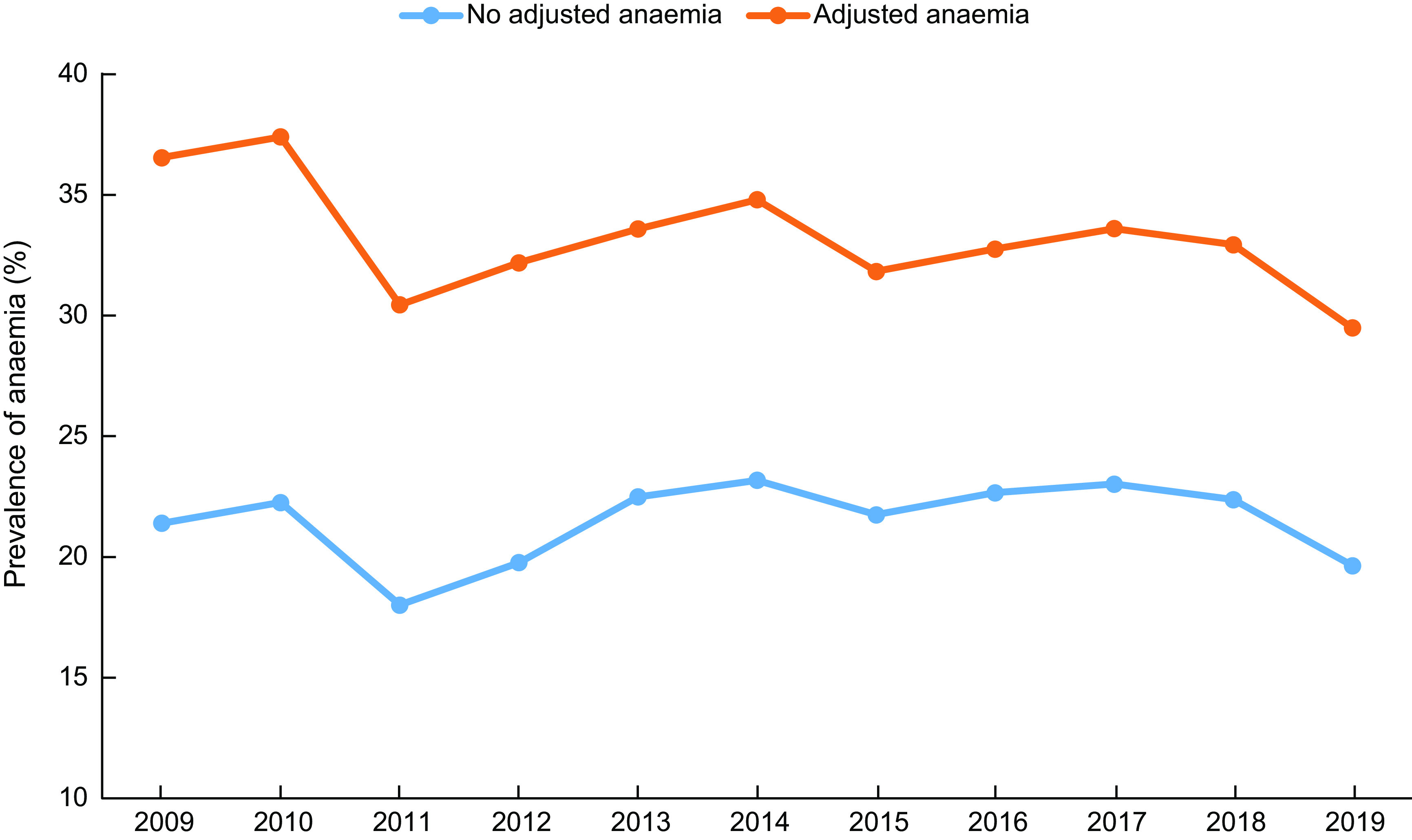
The prevalence of anaemia (%) (adjusted or unadjusted Hb by altitude) in children aged 6–59 months from Peru by survey year

**Fig. 3:**
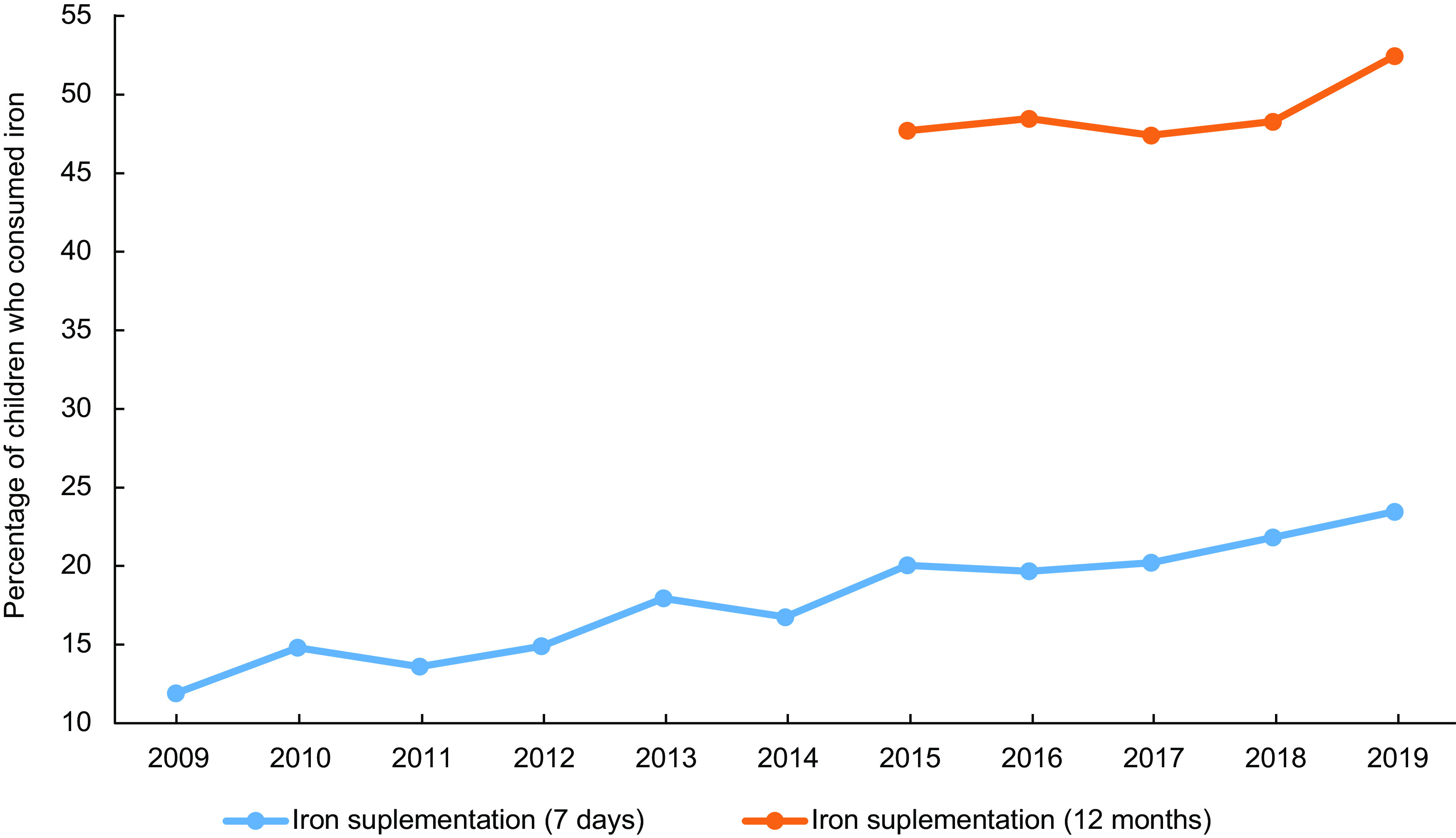
Percentage of children who consumed iron in the last 7 d or 12 months before the survey

The prevalence of anaemia decreased from 2009 to 2011 and increased from 2011 to 2014. From 2014 to 2018 it stagnated, and a reduction was observed from 2018 to 2019 (Fig. 2). The prevalence of anaemia increases 10 points when Hb was corrected to define anaemia at high altitudes. The percentage of children consuming Fe during the last 7 d or 12 months before the survey increased over time (Fig. 3).

The results of the log-binomial regression model showed that Fe supplementation during the 7 d before the survey was associated with episodes of diarrhoea in children (PR = 1·09 ± 0·01; *P* < 0·0001) (95 % CI (1·07, 1·12)). The model controlled for the covariates of drinking water, sanitation service, anaemia, sex, place of residence, region of residence (coast, high altitude and jungle), rotavirus vaccination, mother’s education and age of the children (data not shown). Table 2 shows the results of the two log-binomial regression models used to determine the association between children with recent episodes of diarrhoea and the consumption of Fe supplementation in the last 7 d or 12 months before the survey. The models were controlled for the covariates listed above. Fe supplementation during the last 7 d and the last 12 months before the survey was associated significantly with episodes of diarrhoea in children (PR = 1·09 ± 0·01; *P* < 0·0001 and PR = 1·19 ± 0·03; *P* < 0·0001, respectively).

**Table 2:** The generalised linear model of log-binomial regression of the probability of diarrhoea in children aged 6–59 months

Model	Diarrhoea in children	PR	SE	*P* > |*Z*|	95 % CI	
1	Drinking water					
	No	1·00				
	Yes	0·91	0·03	0·011	0·852	0·979
	Fe supplementation (12 months)					
	No	1·00				
	Yes	1·19	0·03	<0·0001	1·125	1·267
	Anaemia					
	No	1·00				
	Yes	1·17	0·03	<0·0001	1·141	1·288
2	Drinking water					
	No	1·00				
	Yes	0·93	0·02	0·006	0·887	0·980
	Fe supplementation (7 d)					
	No	1·00				
	Yes	1·09	0·01	<0·0001	1·067	1·116
	Anaemia					
	No	1·00				
	Yes	1·20	0·02	<0·0001	1·149	1·253

The General Linear Model is controlled by drinking water, sanitation service, sex, place of residence (rural or urban) and region (coastal, highlands and jungle), rotavirus vaccine, grade of education (no education, elementary, high school and college) and age (6–11, 12–23, 24–35 and 36–59 months).

Drinking water, sanitation service, Fe supplementation, rotavirus vaccination, where 0: No and 1: Yes.

The variable sex corresponds to 0: female and 1: male. Place of residence 0: urban, 1: rural grade of education, 0: no education, 1: primary, 2: secondary and 3: university. Geographic region, 1: Coast, 2: Highlands and 3: Jungle.

Ages in months are categorical variables.

Model 1 = Fe supplementation during 12 months before the survey (sample = 98 321).

Model 2 = Fe supplementation during 7 d prior to the survey (sample = 114 121).

Survey: DHS 2009–2019 (Peru).

Children aged 6–11 months are up to two times more likely to have diarrhoea than children aged 36–59 months (PR = 2·34), while children aged 12–23 months are two and a half times more likely to have diarrhoea (PR = 2·67). Finally, children aged 24–35 months have a 71 % higher risk of diarrhoea (PR = 1·71). The presence of drinking water at home reduced the association with the presence of diarrhoea (PR = 0·91 ± 0·03; *P* < 0·011). Anaemia, male sex, highlands and jungle living, and having a mother without education were also associated with the presence of diarrhoea.

There was not a significant interaction between anaemia and Fe supplementation (during the last 7 d or the last 12 months). The interaction term between anaemia and supplementation in the last 7 d had a *P*-value of 0·96, while the interaction term for anaemia and supplementation in the last 12 had a *P*-value of 0·21.

Fe supplementation during 7 d or 12 months before the survey was associated with a higher prevalence of anaemia (*P* < 0·0001) (Table 3).

**Table 3:** Association between iron supplementation during 7 d or 12 months before the survey and anaemia in children aged 6–59 months

Children	With anaemia		Total	
	*n*	%	*n*	%
With Fe supplementation (7 d)	6646	27·0	24 619	100
Without Fe supplementation (7 d)	18 540	20·7*	89 502	100
With Fe supplementation (12 months)	12 988	25·5	50 937	100
Without Fe supplementation (12 months)	8457	17·8*	47 384	100

Chi-squared test: comparing anaemia with and without Fe supplementation during 7 d prior or 12 months before the survey = *P* < 0·00001.

Anaemia was defined without Hb correction by altitude.

* *P* < 0·00001 respect to the group with Fe supplemmentation.

## Discussion

In the current study, we found that the prevalence of anaemia with Hb altitude correction in Peruvian children aged 6–59 months was higher and remained almost unchanged from years 2009 to 2019. A similar pattern was observed in Bolivian children in which, for over 18 years, the prevalence of anaemia was virtually stagnant. The most consistent characteristic associated with childhood anaemia in Bolivia was diarrhoea in the last 2 weeks^(30)^. According to our results, a similar association between anaemia and diarrhoea was observed for Peru.

This higher prevalence of anaemia is observed even though both countries have developed important intervention programmes with Fe supplementation aimed to reduce anaemia.

The objective of the present investigation was to determine whether Fe supplementation in Peruvian children aged 6 to 59 months is associated with recent episodes of diarrhoea. The results showed that Fe supplementation for 7 d or 12 months before the survey was associated with increased episodes of diarrhoea after controlling for the presence of anaemia. A higher PR was observed when Fe supplementation was given during the last 12 months than during the last 7 d. This would indicate that prolonged consumption, after correction of Fe deficiency, would no longer be beneficial and may put the subject at risk.

This would suggest that children who received supplementation in the last 12 months and who present diarrhoea could be receiving excess Fe. The excessive Fe concentrations not being absorbed in the duodenum would enter the colon and be used by pathogenic bacteria to the detriment of commensal bacteria^(31)^. The amount of Fe in supplementation modifies the composition of the infant gut microbiota and increases faecal calprotectin levels, a marker of gut inflammation associated with an increased incidence of diarrhoea^(23)^.

According to our results, the PR for diarrhoea was higher when children received Fe supplementation during the last 12 months than during the last 7 d. This suggests that chronic exposure may increase the amount of Fe in the large intestine, increasing the chance of dysbiosis. A study in healthy weaning piglets showed increased diarrhoeal incidence associated with high dietary Fe intake, changing the intestinal immune response-associated gene expression and shifting the gut microbiota composition^(32)^.

Inflammation has infectious and non-infectious causes. Infectious diseases may be due to bacteria, parasites and viruses. In some settings, parasites such as soil-transmitted helminths are endemic. In the Peruvian jungle region, populations have a high prevalence of soil-transmitted helminth, anaemia and malnutrition^(33)^. Soil-transmitted helminth probably causes mild to severe diarrhoea, weakness, intestinal blood loss, and impaired cognitive development and growth among others^(34)^. Compared to data from the coast and high altitudes, the PR for diarrhoea is higher in children living in the Peruvian jungle.

Anaemia was also associated with diarrhoea. Although the data do not allow us to determine the proportion of anaemia attributable to Fe, data from the literature also suggest an association between duodenal dysbiosis, iron deficiency anaemia and diarrhoea^(35)^. However, inflammatory anaemia could be also associated with diarrhoea^(36)^.

Additionally, the results of the analysis show that having sanitation service facilities decreases the percentage of anaemic children with diarrhoea episodes associated with Fe supplementation in the last 12 months. Households with sanitation facilities allow children to have proper hygiene, reducing the risk of ingesting parasites and faecal bacteria, thereby limiting the risk of infection and the consequence of a local and subsequent systemic inflammation^(37)^.

Access to safe water and basic sanitation and hygiene facilities (WASH) are important for childhood health. In such a sense, low change levels in overall WASH access may reduce the prevalence of diarrhoea in low- and middle-income countries such as Peru^(38)^.

We observed that Peruvian children living in houses with sanitation had a lower incidence rate of diarrhoea than those living in houses without sanitation. This is consistent with findings that a high prevalence of the diarrhoeal disease is observed among children in schools without WASH interventions compared with schools having WASH interventions^(39)^.

Our study also showed that in children, male sex, with poorly educated mothers, and younger age were associated with a higher risk of diarrhoea. Previous studies showed similar results with mothers’ education and recommend that female education should be encouraged to enhance the survival of neonates and infants^(40)^. Higher rates of diarrhoea in younger children from 6 to 35 months than in older children are likely due to the weaning occurring at 6 months in most children. It has been suggested that health education programmes should be directed towards mothers to improve rates of breast-feeding, weaning practices, food hygiene and childcare. Special consideration and support must be given to working mothers^(41)^.

In our study, people who had diarrhoea presented worse living conditions, a higher percentage of anaemia, and used Fe supplementation. There is not a policy in Peru to encourage Fe supplementation in people with the worst socio-economic conditions. The policy of Fe supplementation is unique to the whole country.

Based on previous findings and our results, it is clear that children should be supplemented with F only when a Fe deficiency is detected. This intervention is important because Fe fulfils important functions in the body. Therefore, the measurement of serum ferritin as a marker of Fe status should be implemented as a strategy of the governments. Conversely, excess Fe in a child’s gut, who is not Fe-deficient, will be harmful^(42)^.

It is important to consider different variables that can increase or decrease the risk of diarrhoea production in the context of excess Fe supplementation. These include the availability of drinking water and sanitation services for safe excreta disposal.

Reducing Fe deficiency has beneficial effects on neurodevelopment, but Fe excess in the gut may have adverse functional effects, including diarrhoea and even poor neurodevelopment^(43)^.

One limitation of the study is that ferritin data for the classification of iron deficiency anaemia were not available. This would allow estimating how much of the anaemia in Peru is due to Fe deficiency. It would be important if the Peruvian government include ferritin measurement as a strategy to fight against anaemia. In Peru, the only diagnosis criteria used to define anaemia was the Hb measurement. The second limitation is that our study is from a cross-section design, but data suggest that the high prevalence of anaemia in Peru results from other causes different from Fe deficiency. Since excess Fe could be harmful, we must be careful when universal treatment with Fe supplementation and Fe fortification is promoted.

The strength of our research is that it includes information for the assessment of anaemia, Fe supplementation and diarrhoea data over time in representative samples for all the country. The implications of this study would be that actually Fe treatment has more harm than benefits.

According to the time trend in Peru, anaemia was reduced significantly from 2000 to 2010 but from 2011 to our days, anaemia prevalence was maintained almost without changes and the goal for the reduction of anaemia in Peru, according to the government, has not been accomplished yet. For example, the goal for the anaemia rate was expected to be 23·8 % for 2020, but the anaemia rate observed was 40 %. Then, within Peru, the time trends of Fe efficacy do not suggest that intervention is working.

Systematic studies show that when there is iron deficiency anaemia, Fe supplementation intervention increases Hb and ferritin levels^(44)^ and reduces rates of anaemia and Fe deficiency in children^(45)^. The risk of anaemia was reduced with Fe alone, Fe-folic acid, multimicronutrients supplementation, multimicronutrient powder, targeted fortification and large-scale fortification^(46)^. However, the benefits of this intervention as a child survival strategy or for developmental outcomes are unclear^(45)^.

In children aged 4–23 months, daily Fe supplementation effectively reduces anaemia. However, the adverse effect profile of Fe supplements and effects on development and growth is uncertain. Vomiting and fever were more prevalent in children receiving Fe^(47)^. According to another study, the scientific evidence is insufficient to recommend oral Fe therapy to children with non-anaemic Fe deficiency^(48)^.

Fortification of wheat flour^(49)^ or rice^(50)^ with Fe alone have little effect on anaemia and probably makes little or no difference in the risk of presenting Fe deficiency, and there is uncertainty that interventions may increase the mean Hb concentrations in the general population of children older than 2 years of age.

## Conclusions

In conclusion, the present study showed that Fe supplementation during the 7 d or 12 months before the survey is associated with the risk of diarrhoea in children aged 6–59 months in Peru.
